# Enhancing patient safety in the emergency care of poisoned patients: a participatory systems approach

**DOI:** 10.1093/intqhc/mzag050

**Published:** 2026-04-27

**Authors:** Mecit Can Emre Simsekler, Firda Rahmadani, Nahed Ali AlRaeesi, Mozah Mohamed Alsereidi

**Affiliations:** Department of Management Science & Engineering, Khalifa University of Science & Technology, Abu Dhabi, UAE; Department of Management Science & Engineering, Khalifa University of Science & Technology, Abu Dhabi, UAE; Department of Health Abu Dhabi, Abu Dhabi, UAE; Department of Health Abu Dhabi, Abu Dhabi, UAE

**Keywords:** patient safety, emergency care, poisoning, human-centered design, participatory systems approach, process mapping, quality improvement

## Abstract

**Background:**

Poisoning is a leading cause of injury-related mortality globally, with emergency departments (EDs) playing a critical role in managing these cases. However, treating poisoned patients in EDs presents unique challenges, including variations in clinical presentations, delays in toxicology testing, and communication breakdowns among healthcare professionals.

**Methods:**

This study aimed to develop practical guidance for managing poisoned patients in EDs by engaging multidisciplinary stakeholders using a participatory systems design approach. Structured brainstorming sessions were conducted with 38 healthcare professionals, including toxicologists, emergency nurses, and physicians, organized into six multidisciplinary teams. The Improvement Canvas, developed at the Cambridge Engineering Design Centre, was used to guide participants in identifying key challenges, mapping system dynamics, defining core problems, and proposing targeted solutions. The guidance was refined through multiple iterations and shared with representatives from the Department of Health Abu Dhabi for feedback. Additionally, process maps were created to facilitate the implementation of recommendations across regional hospitals.

**Results:**

Participants identified several critical areas for improvement, including standardized workflows for triage, resuscitation, and toxicology testing; enhanced mental health support; and improved interdepartmental communication. The resulting framework emphasizes continuous monitoring, real-time information sharing, and cross-functional collaboration among healthcare teams. Feedback from the Department of Health validated the practical relevance and feasibility of the proposed strategies.

**Conclusion:**

By integrating frontline expertise and systems thinking, this study delivers evidence-based recommendations to standardize care and enhance patient safety in emergency settings. The developed framework supports streamlined emergency care processes by fostering effective teamwork and incorporating perspectives from mental health and toxicology. Implementing these strategies can improve care efficiency and ensure high-quality outcomes for poisoned patients in EDs.

## Introduction

Poisoning occurs when exposure to toxic substances, such as chemicals, medications, biological agents, environmental or occupational toxins, or illicit drugs, results in harmful effects. It is a significant global health concern, contributing to both morbidity and mortality [1]. Although largely preventable, poisoning remains a leading cause of injury-related deaths worldwide, with fatality rates varying across cultural and societal contexts [2]. For instance, in the USA, poisoning is the leading cause of injury-related mortality, accounting for over 40 000 deaths annually [1]. Drugs contribute to 90% of these fatalities, with drug-related poisoning deaths rising significantly in recent years [3]. Emergency departments (EDs) play a crucial role in managing poisoning cases, as they serve as the first point of care for poisoned patients [1].

Managing poisoned patients in EDs presents unique challenges due to the variability in clinical effects, which depend on factors such as the toxic dose, duration of exposure, and the patients’ preexisting health conditions [4]. Early recognition, timely diagnostic testing, and appropriate supportive care are critical for improving patient prognosis. Additionally, understanding the proper use of antidotes, including their indications and contraindications, is essential for effective management in poisoning emergencies [5].

Ensuring patient safety in poisoning cases requires an understanding of toxicology principles and the ability to recognize clinical signs and symptoms [6]. Effective management strategies include a thorough history, a detailed physical assessment, and adherence to established clinical protocols. Immediate resuscitation, airway stabilization, and circulatory support are top priorities [7]. For example, an observation period of 6 hours is often recommended to rule out severe toxicity before discharge [8]. While early decontamination and antidote administration are crucial, not all toxins have a specific antidote [9]. Continuous monitoring of vital signs, neurological assessments, and laboratory testing helps ensure accurate diagnosis and appropriate intervention [10]. Given these complexities, hospitals must adopt best practices and foster a culture of safety to minimize medical errors in the management of poisonings [11]. Standardizing poisoning management in emergency settings is therefore key to ensuring timely and effective care.

Collaboration among healthcare professionals is critical to ensuring an effective response to poisoning emergencies [12]. A multidisciplinary approach, including toxicologists, emergency medicine specialists, and laboratory personnel, enhances diagnostic accuracy, treatment effectiveness, and resource efficiency [13]. Previous studies emphasize the importance of improving emergency medicine residents’ confidence in managing poisonings, as this directly impacts patient safety [13]. However, challenges in collaboration arise due to varying levels of toxicology awareness, inconsistent application of management protocols, and communication barriers [14]. These issues can delay response times and hinder treatment effectiveness. Addressing these gaps requires standardized training on individualized management approaches, enhanced interprofessional communication, and clear clinical guidelines for acute poisoning cases [15]. Additionally, fostering a culture of teamwork and shared decision-making can improve risk assessment and ensure optimal patient outcomes [16, 17].

Despite the urgency of poisoning emergencies, standardized, consensus-driven protocols for ED management remain lacking, particularly in addressing social and cultural differences. Variability in clinical decision-making, differences in toxicology knowledge, and inconsistent treatment approaches contribute to suboptimal outcomes. Establishing clear guidelines and structured care pathways is essential to enhancing coordination, reducing treatment delays, and improving overall patient safety [18].

This study aims to address these challenges by integrating diverse perspectives from healthcare stakeholders to develop practical guidance for the management of poisoned patients in EDs. A systems approach is most effective when all relevant stakeholders actively contribute, and their input is incorporated into system design [19, 20]. Although engaging stakeholders in healthcare improvement can be challenging, evidence suggests that their participation enhances service responsiveness and efficiency [21]. Using a participatory systems design approach, this study engaged toxicologists, emergency medicine specialists, pharmacists, and other key professionals to collaboratively identify challenges, define priorities, and propose structured management strategies. By leveraging their collective expertise, the study sought to promote a shared understanding of poisoning management and enhance the standardization of care.

A key contribution of this research is its emphasis on participatory engagement, ensuring that the proposed guidance reflects real-world clinical experiences and operational needs. This approach provides a structured mechanism for stakeholders to communicate and prioritize key issues, leading to meaningful improvements in ED processes. By integrating frontline experiences with a systems-thinking perspective, this study aims to bridge gaps in poisoning management, promote interprofessional collaboration, and ultimately enhance patient outcomes in emergency settings.

## Materials and methods

This study employed a participatory systems approach to develop guidance for managing poisoned patients in emergency settings that could later be evaluated in a clinical care setting. It involves actively engaging stakeholders across a system to collaboratively analyze problems, map interactions, and co-design feasible solutions. This approach is grounded in the principles of systems thinking (recognizing that interdependent components shape clinical processes and outcomes) and participatory design, which values the expertise of frontline users in informing improvements to complex workflows [22]. A brainstorming session was held with 38 participants, divided into six multidisciplinary teams comprising healthcare professionals from various settings across Abu Dhabi, United Arab Emirates.

Participants included carers, emergency nurses, toxicologists, pharmacists, physicians, and healthcare managers. They were primarily selected through purposeful sampling, the sole sampling strategy to ensure representation of key roles and specialties, such as toxicologists, whom the project team identified as particularly valuable. The aim of this sampling approach was not statistical representativeness but rather to ensure multidisciplinary coverage and capture diverse system-level perspectives relevant to emergency toxicology care. Although the sample does not encompass all healthcare professionals working in the region, it includes the principal stakeholders who participate in clinical decision-making and operational workflows for poisoned patients.

To systematically structure brainstorming sessions, the Improvement Canvas, developed by the Healthcare Design Group at the Cambridge Engineering Design Centre, was utilized [23, 24]. This tool helped teams map their current understanding of the system, identify key challenges, and define the necessary scope for change. At the beginning of each improvement stage, the teams reaffirmed their canvas entries to ensure clarity on the current state, desired outcomes, and pathways to bridge existing gaps. Each entry captured team insights, enabling a structured approach to problem definition and solution development. Individual contributions were not recorded verbatim, as the focus was on compiling shared outputs rather than documenting attributable statements.

The Improvement Canvas was used to guide participants through a structured problem analysis and solution generation process. To support consistent and focused exploration of system dynamics, each group was provided with a set of clinically typical poisoning scenarios (e.g. an unconscious patient with suspected overdose, an agitated patient with unknown ingestion). These scenarios served as anchors for the mapping and scoping exercises, helping participants identify workflow steps, bottlenecks, information needs, and interdepartmental dependencies. During the system-mapping phase, scenarios enabled participants to trace how a representative case would move through triage, resuscitation, toxicology testing, mental health assessment, and disposition. This ensured that the Improvement Canvas outputs reflected realistic clinical pathways and highlighted practical challenges encountered in routine emergency care. The scenarios also helped define the scope of the problem by focusing discussions on specific patient trajectories rather than abstract generalizations.

To ensure consistency across teams, the scenario and scope were defined before the workshop, providing a common starting point for all participants. The following was provided as the scenario:

“*A young patient is rushed to the ED after ingesting a significant amount of an unknown substance in what appears to be a potential suicide attempt. The patient is conscious but confused and uncooperative. Initial assessments by the ED staff are inconclusive, and it is unclear what toxins the patient may have consumed. As time is critical, a systematic approach must be adopted to minimize the risk of missing potential toxicities*.”

We have also provided the scope as outlined below:

“*To ensure that healthcare professionals can work together to minimize the risk of misdiagnosis and ensure a standardized, systematic approach in assessing patients suspected of poisoning, particularly those involving suicide attempts*.”

After clarifying the scenario and completing the (i) Agree to the scope stage, the process followed the steps outlined in the Improvement Canvas: (ii) Understand the Context, (iii) Define the Problem, (iv) Identify Stakeholders, (v) Develop Solutions, (vi) Collect Evidence, (vii) Build the Team, (viii) Make the Case, and (ix) Manage the Plan.

Following the brainstorming sessions, the research team compiled and synthesized the proposed solutions into a comprehensive framework. The preliminary results were shared with stakeholders at the Department of Health Abu Dhabi for feedback and refinement. Through multiple rounds of revisions and short discussion sessions, the guidance was finalized, accompanied by a series of process maps to enhance healthcare providers’ and stakeholders’ understanding of patient safety management for poisoned patients in emergency settings. These maps included a *Stakeholder Diagram* to identify key participants in the process, an *Information Diagram* to outline the hierarchy of information and materials, a *Communication Diagram* to illustrate the flow of information and materials between individuals and processes, and a *Flow Diagram* to show the sequence and parallel activities within the process [25]. These maps were designed to visually represent the interconnections and links [26], facilitating further analysis and stakeholder validation of the proposed process. This iterative approach ensured the guidance was both evidence-informed and practically applicable to hospitals in Abu Dhabi.

Evidence supporting the perceived usefulness of the framework was derived from qualitative data generated during the participatory design sessions. These included structured brainstorming outputs, systems mapping observations, discussion notes, and group consensus statements. In addition, formal feedback from the Department of Health, Abu Dhabi was collected to assess the feasibility and practical relevance of the proposed processes. No quantitative outcome measures or implementation metrics were collected, as the framework was not deployed in real-time clinical workflows during this study.

## Results

The participants began with the “Understand the Context” stage, in which they explored the broader healthcare ecosystem involved in poisoning case management. They identified key stakeholders, including ED staff, mental health professionals, toxicology labs, and social services, all of whom play a crucial role in patient care. The discussion highlighted systemic challenges, including time-sensitive toxicology screening, coordination difficulties among healthcare providers, and the need for improved communication with external agencies, such as law enforcement. Additionally, participants recognized the diversity of patients presenting with suspected poisoning, noting that many may have underlying mental health conditions, language barriers, or complex medical histories. Addressing these factors was considered vital for delivering effective and equitable care.

The next stage was titled “Define the Problem,” in which participants identified critical barriers affecting the timely diagnosis and management of poisoning cases. The most pressing concerns included limited access to toxicology testing, inadequate training for triage staff, and communication breakdowns across facilities. Other challenges discussed included logistical delays in toxicology sample transport, insurance-related constraints affecting test reimbursements, and cultural stigmas surrounding suicide attempts that might deter individuals from seeking immediate care. The group agreed that the highest priority should be ensuring patient safety through rapid diagnosis, clear communication, and improved access to mental health support.

The next stage was “Identify the Stakeholders,” during which participants mapped the various individuals and institutions involved in managing poisoning cases. Key stakeholders identified included patients and their families, ED healthcare providers (such as nurses, physicians, toxicologists, and paramedics), decision-makers from the DoH Abu Dhabi, social service workers, insurance companies, pharmacists, laboratory technicians, and law enforcement personnel. The groups emphasized the need for a collaborative and multidisciplinary approach to address gaps in care and streamline processes for managing poisoning incidents.

The next stage was “Develop the Solution,” where participants proposed several key interventions to improve patient outcomes. These included establishing a standardized workflow for poisoning cases, ensuring early resuscitation and risk assessment, and providing specialized training for healthcare providers on toxicology and mental health protocols. Additional recommendations focused on expanding access to toxicology testing across all EDs, improving communication among emergency staff, laboratories, and families, and optimizing logistics to ensure timely processing of toxicology samples. By implementing these strategies, hospitals could enhance efficiency and ensure prompt, high-quality care for poisoned patients.

The next stage was “Collect the Evidence,” where the participants underscored the importance of evaluating the effectiveness and safety of the proposed interventions. They suggested tracking key performance indicators, including mortality and morbidity rates among poisoning cases, turnaround times for toxicology test results (targeting <2 hours), ICU transfers, readmission rates, and the outcomes of mental health referrals. Additionally, they highlighted potential risks, including misdiagnosis due to delayed testing and insufficient psychiatric support, and emphasized the need for continuous monitoring and quality improvement initiatives.

The follow-up stage was “Build the Team,” where the participants recognized that sustained improvement in poisoning case management required strong collaboration among key stakeholders. They emphasized the importance of engaging the Department of Health Abu Dhabi, hospital administrators, ED staff, toxicologists, psychiatrists, lab technicians, social workers, and police representatives to develop and implement standardized protocols. Establishing a multidisciplinary team dedicated to ongoing evaluation and adaptation of these protocols was considered essential for long-term success.

The next stage was “Make the Case,” during which participants recommended conducting comprehensive root-cause analyses of poisoning cases to identify safety concerns and prevent recurrence. They also emphasized the need for accessible medical records across healthcare facilities to enhance care coordination and reduce diagnostic errors. Additionally, they advocated streamlining hospital workflows to minimize delays and optimize resource utilization, ensuring that poisoned patients receive timely and effective treatment.

Finally, in the last stage, “Manage the Plan,” the participants outlined a strategy for implementing and sustaining the proposed improvements. They recommended introducing a DoH-mandated standard for managing poisoned patients, supported by regular audits to assess compliance and effectiveness. Gathering feedback from frontline healthcare providers and patients was also deemed crucial for refining protocols. Furthermore, they proposed establishing a dedicated task force to monitor progress, address emerging challenges, and ensure continuous improvement in poisoning case management.

Overall, the structured discussions and collaborative efforts in the workshop enabled participants to develop a practical, evidence-based approach to improving the management of poisoning cases in EDs. While the framework was not applied to actual patient cases, participants used a series of hypothetical, but clinically typical, poisoning scenarios to test the practicality and coherence of the workflow steps. These exercises helped refine the framework but did not constitute implementation in a live clinical environment. The proposed interventions, if effectively implemented, have the potential to enhance patient safety, streamline workflows, and improve access to critical toxicology and mental health resources.

Participants consistently reported that the framework clarified workflow steps, improved shared understanding of roles, and highlighted critical system bottlenecks. These perceptions were evident in group discussions and outputs from the Improvement Canvas exercises. Representatives from the Department of Health affirmed the framework’s alignment with regional priorities and its feasibility for future implementation. While these qualitative data indicate strong perceived usefulness, they do not constitute evidence of clinical impact.

**Figure 1:**
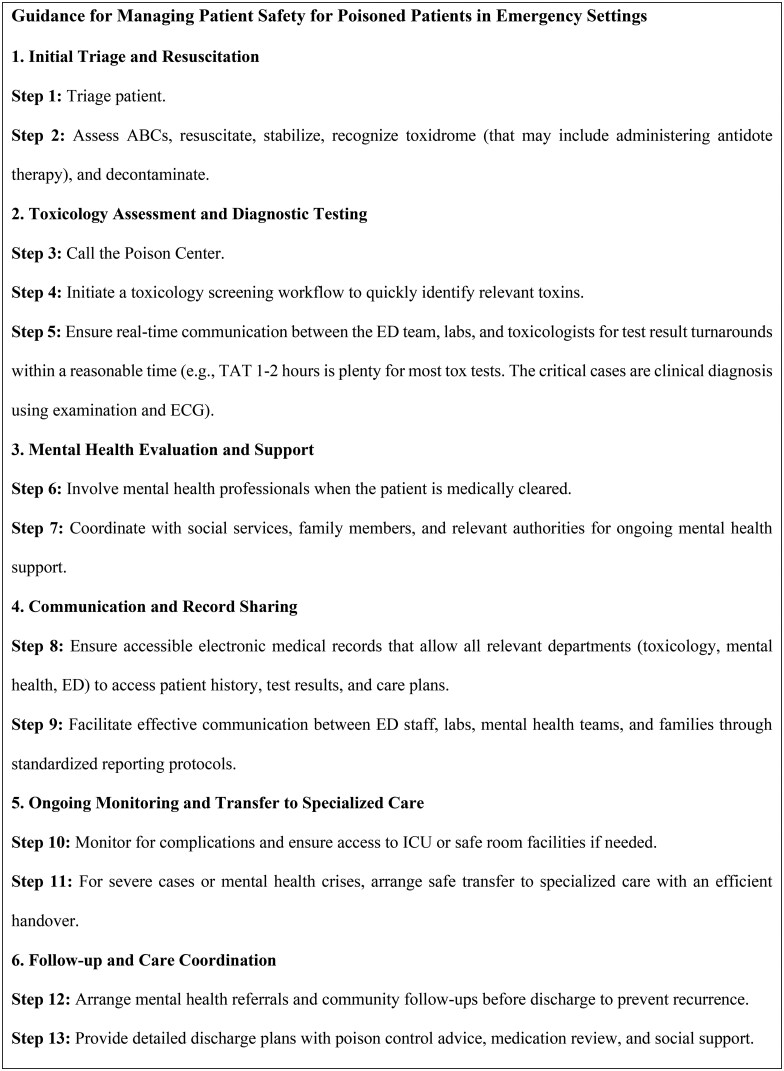
Guidance for managing patient safety for poisoned patients in emergency settings.

Following the workshop, the compiled notes were shared with the Department of Health Abu Dhabi to provide a comprehensive overview of patient safety management for poisoned patients in emergency settings. Based on the gathered information and an iterative validation process, the following guidance (see Fig. 1) was recommended for dissemination to hospitals to establish a streamlined, standardized approach to managing poisoned patients in emergency services.

**Figure 2:**
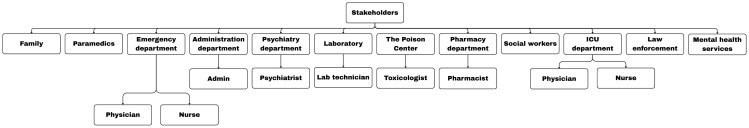
Stakeholder Diagram.

**Figure 3:**
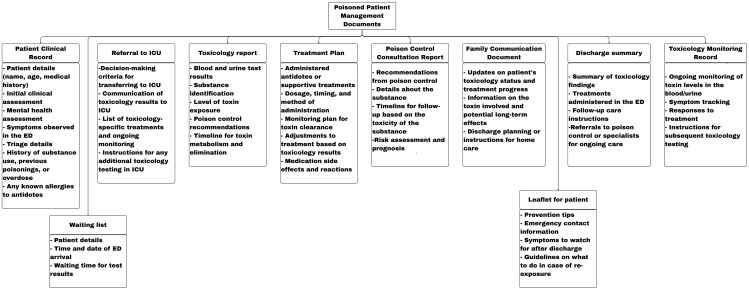
Information Diagram.

**Figure 4:**
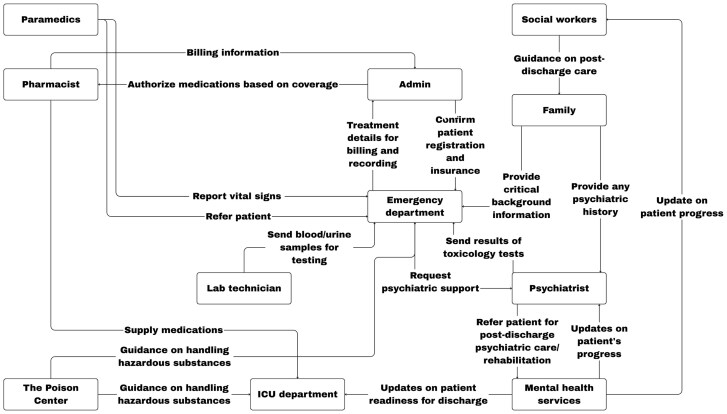
Communication Diagram.

**Figure 5:**
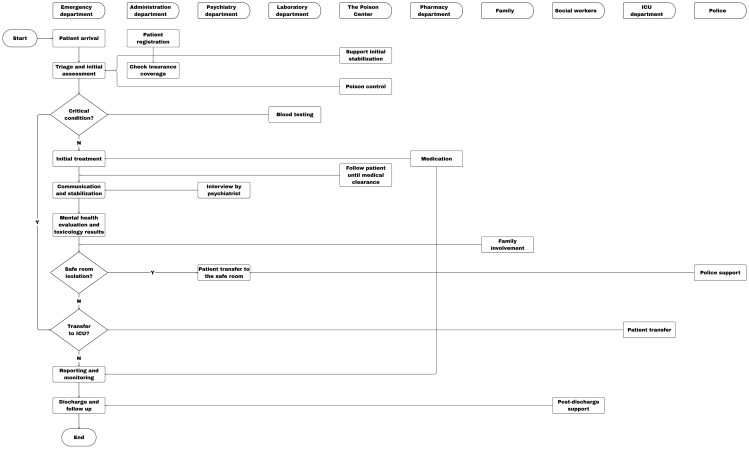
Flow Diagram.

Additionally, several process maps (see Figs 2–5) were accompanied by guidance to enhance understanding and visually represent the various structures and behaviors within the process.

Each diagram served a specific purpose in mapping key participants, information flow, communication pathways, and the sequence of activities involved in patient care. The Stakeholder Diagram identified the key participants and organizational divisions involved in the management process. This included critical roles such as toxicologists at the Poison Center, ED physicians, laboratory technicians, psychiatrists, and other healthcare professionals. By mapping these stakeholders, the diagram clarified each participant’s responsibilities and highlighted the collaborative efforts required across multiple departments to ensure effective patient care. This visual representation was essential in understanding how different teams interact and contribute to the patient management process.

The Information Diagram outlined the hierarchy and flow of essential information and materials used throughout the patient care process. Key documents were identified, including the toxicology report, family communication document, patient information leaflet, and discharge summary. This diagram also details when and how these materials are generated, shared, and utilized at various stages of patient care. By organizing the flow of information, the diagram facilitated a clearer understanding of the documentation process and ensured that all critical data points were captured and communicated effectively.

The Communication Diagram illustrated the flow of information and materials between individuals and processes. It detailed key communication pathways, such as the exchange of information between the ED and laboratory technicians for toxicology testing and between the ED and psychiatrists for mental health assessments. This diagram highlights both formal and informal communication channels, ensuring that all critical information flows seamlessly across departments. By mapping these interactions, the diagram helped identify potential communication gaps and areas for improvement in real-time information sharing.

The Flow Diagram depicted the sequence and parallel activities involved in the patient journey, from their arrival at the ED to discharge and follow-up care. It provided a step-by-step visualization of the patient’s movement through the healthcare system, capturing key stages such as initial assessment, laboratory testing, specialist consultations, treatment administration, discharge planning, and post-discharge follow-up. This diagram was particularly useful for identifying bottlenecks, optimizing workflow efficiency, and ensuring that patient care processes were both timely and standardized.

Together, these four diagrams provided a holistic view of the patient safety management process for poisoned patients in emergency settings. They facilitated a deeper understanding of the roles, communication flows, information pathways, and procedural sequences, supporting the development of a more efficient, standardized, and patient-centered approach to managing poisoned patients.

## Discussion

### Statement of principal findings

This study highlights the significance of utilizing a participatory systems approach to improve the management of poisoned patients in emergency care settings. By engaging multidisciplinary teams, we identified critical challenges and proposed actionable solutions that streamline patient management processes, enhance patient safety, and optimize ED workflows. Key findings included the urgent need for standardized to poisoning diagnosis, early resuscitation, and coordinated communication across care teams. Delays in toxicology testing and limited access to mental health services were also recognized as major barriers.

### Strengths and limitations

A major strength of this study lies in its participatory and multidisciplinary design, which enabled the inclusion of diverse perspectives from toxicologists, emergency medical staff, mental health professionals, and lab technicians. This diversity helped reveal practical challenges and identify feasible solutions. The use of the Improvement Canvas and the development of process maps further strengthened the design by facilitating structured problem-solving and visualizing complex care processes. However, the study is not without limitations. It was conducted in a specific regional context, which may limit the generalizability of the findings to other healthcare systems. Additionally, while the guidance developed was shared with health authorities, the real-world impact of these recommendations has not yet been evaluated through implementation studies or longitudinal data. Because this study involved 38 purposively selected participants, the findings should be interpreted in terms of their transferability rather than generalizability. The participatory systems design approach prioritizes depth of insight into workflow challenges and system dynamics over broad population representation. Therefore, the applicability of the proposed framework may vary in settings with different organizational structures, resource availability, or care pathways. Nonetheless, the inclusion of diverse clinical roles and validation from the Department of Health enhances the recommendations’ relevance in similar emergency care contexts. The framework has not yet been implemented or evaluated in routine clinical practice, and no patient cases were managed using the proposed processes during the study period. As such, the findings reflect a co-design and validation phase rather than a clinical effectiveness assessment. Future work will involve pilot implementation and outcome evaluation across selected EDs.

### Interpretation within the context of the wider literature

The findings of this study are consistent with existing literature that emphasizes the importance of early intervention [15], integrated mental health support [27], and effective communication in managing poisoned patients [28]. Previous research has highlighted similar issues, such as the need for standardized workflows and the benefits of multidisciplinary collaboration in emergency settings. This study contributes to the broader evidence base by offering a structured and participatory method [21] for identifying system-level challenges and proposing solutions tailored to frontline experiences. The integration of mental health considerations into poisoning management also reflects a growing awareness in the literature of the overlap between physical and psychological health in emergency care [29].

### Implications for policy, practice, and research

The proposed framework offers practical implications for hospital administrators, policymakers, and emergency care practitioners. Hospitals can use the guidance to standardize care protocols, improve toxicology test turnaround times, and enhance mental health support for poisoning cases. Further, the process maps and structured communication strategies can be used for staff training and performance evaluation. For policymakers, the findings support the development of region-wide protocols for poisoning management and highlight the need for investments in diagnostic and mental health services.

Future research should focus on implementing these recommendations, particularly by evaluating their impact on patient outcomes, interdepartmental collaboration, and overall system performance in emergency settings.

## Conclusions

The participatory systems approach adopted in this study has yielded actionable guidance for improving the management of poisoned patients in EDs. The final framework emphasizes early diagnosis, interdisciplinary collaboration, efficient communication, and integration of mental health. By adopting these strategies, hospitals can improve care coordination, enhance patient safety, and ensure better health outcomes. Further research is needed to validate the framework in diverse healthcare settings and to assess its long-term impact on patient care and ED performance.

## Data Availability

All findings from the workshop described in this study are presented within the manuscript. Qualitative results are integrated into Figs 1–5.

## References

[1] Warner M , Chen LH, Makuc DM, et al. Drug Poisoning Deaths in the United States, 1980–2008, no. 81. NCHS Data Brief, 2011, Hyattsville, MD: NationalCenter for Health Statistics. 22617462

[2] Mintegi S , Azkunaga B, Prego J, et al; Pediatric Emergency Research Networks (PERN) Poisoning Working Group. International epidemiological differences in acute poisonings in pediatric emergency departments. Pediatr Emerg Care 2019;35:50–7. 10.1097/PEC.0000000000001031 28121975

[3] Chen LH , Hedegaard H, Warner M. Drug-poisoning deaths involving opioid analgesics: United states, 1999-2011. *NCHS Data Brief* 2014;1–8. 25228059

[4] Rasimas JJ , Carter GL. Psychiatric issues in the critically poisoned patient. In: Brent J, Burkhart K, Dargan P et al (eds.), Critical Care Toxicology. Cham: Springer International Publishing, 2016, 1–41. 10.1007/978-3-319-20790-2_44-1

[5] Marraffa JM , Cohen V, Howland MA. Antidotes for toxicological emergencies: a practical review. Am J Health Syst Pharm 2012;69:199–212. 10.2146/ajhp110014 22261941

[6] Humphries C , Eddleston M, Dear J. The emergency treatment of poisoning. Medicine (Baltimore) 2024;52:56–61. 10.1016/j.mpmed.2023.10.004

[7] Thim T , Krarup NHV, Grove EL et al Initial assessment and treatment with the airway, breathing, circulation, disability, exposure (ABCDE) approach. Int J Gen Med 2012;5:117–21. 10.2147/IJGM.S28478 22319249 PMC3273374

[8] Moreira M , Buchanan J, Heard K. Validation of a 6-hour observation period for cocaine body stuffers. Am J Emerg Med 2011;29:299–303. 10.1016/j.ajem.2009.11.022 20825819 PMC3000892

[9] Kobylarz D , Noga M, Frydrych A et al Antidotes in clinical toxicology—critical review. Toxics 2023;11:723. 10.3390/toxics11090723 37755734 PMC10534475

[10] Titi D. Management of acute poisonous cases in the emergency department. Sch Acad J Biosci 2023;11:27–32. 10.36347/sajb.2023.v11i01.005

[11] Simsekler MCE , Qazi A, Ozonoff A. Exploring the role of safety culture dimensions in patient safety using a Bayesian belief network model. Saf Sci 2025;186:106817. 10.1016/j.ssci.2025.106817

[12] Saint-Pierre C , Herskovic V, Sepúlveda M. Multidisciplinary collaboration in primary care: a systematic review. Family Practice 2018;35:132–41. 10.1093/fampra/cmx085 28973173

[13] Smith SW , Farmer BM. Toxicology in the service of patient and medication safety: a selected glance at past and present innovations. J Med Toxicol 2015;11:245–52. 10.1007/s13181-015-0470-3 25804670 PMC4469728

[14] Rawlinson C , Carron T, Cohidon C et al An overview of reviews on interprofessional collaboration in primary care: barriers and facilitators. Int J Integr Care 2021;21:32. 10.5334/ijic.5589 34220396 PMC8231480

[15] Al-Jelaify M , AlHomidah S. The individualized management approach for acute poisoning. Adv Pharmacol Pharm Sci 2021;2021:9926682–5. 10.1155/2021/9926682 34056610 PMC8133860

[16] Kaya GK , Ustebay S, Nixon J et al Exploring the impact of safety culture on incident reporting: lessons learned from machine learning analysis of NHS England staff survey and incident data. Saf Sci 2023;166:106260. 10.1016/j.ssci.2023.106260

[17] Simsekler MCE , Rodrigues C, Qazi A et al A comparative study of patient and staff safety evaluation using tree-based machine learning algorithms. Reliab Eng Syst Saf 2021;208:107416. 10.1016/j.ress.2020.107416

[18] Kaya GK. A system safety approach to assessing risks in the sepsis treatment process. Appl Ergon 2021;94:103408. 10.1016/j.apergo.2021.103408 33711556

[19] Hettinger LJ , Kirlik A, Goh YM et al Modelling and simulation of complex sociotechnical systems: envisioning and analysing work environments. Ergonomics 2015;58:600–14. 10.1080/00140139.2015.1008586 25761227 PMC4647651

[20] Waterson P , Catchpole K. Human factors in healthcare: welcome progress, but still scratching the surface, viewpoint. BMJ Qual Saf 2016;25:480–4. 10.1136/bmjqs-2015-005074 26685148

[21] Jun GT , Canham A, Altuna-Palacios A et al A participatory systems approach to design for safer integrated medicine management. Ergonomics 2018;61:48–68. 10.1080/00140139.2017.1329939 28506152

[22] Hardie GJ. Community participation based on three-dimensional simulation models. Des Stud 1988;9:56–61. 10.1016/0142-694X(88)90026-9

[23] Cambridge Engineering Design Centre. Improvement canvas. In: *Improving Improvement: A Toolkit for Engineering Better Care*, 2025. https://www.iitoolkit.com/resources/Worksheets_2_16_Improvement_Canvas.pdf. Accessed on 08.02.2025

[24] Cambridge EDC. Improving Improvement: A Toolkit for Engineering Better Care. Cambridge: Cambridge Engineering Design Centre, 2025. www.iitoolkit.com

[25] Simsekler MCE , Ward JR, Clarkson PJ. Evaluation of system mapping approaches in identifying patient safety risks. Int J Qual Health Care 2018;30:227–33. 10.1093/intqhc/mzx176 29346654 PMC6104811

[26] Wheway JL , Jun GT. Adopting systems models for multiple incident analysis: utility and usability. Int J Qual Health Care 2021;33:mzab135. 10.1093/intqhc/mzab135 34508632

[27] Gill AD , McCuin T, Maron M. Program development of integrated psychological services for hospitalized patients with intravenous drug use histories. J Clin Psychol Med Settings 2020;27:22–30. 10.1007/s10880-019-09616-4 30949791

[28] Ellington L , Matwin S, Jasti S et al Poison control center communication and impact on patient adherence. Clin Toxicol (Phila) 2008;46:105–9. 10.1080/15563650701338914 18259957 PMC3167482

[29] Grimholt TK , Bjornaas MA, Jacobsen D et al Treatment received, satisfaction with health care services, and psychiatric symptoms 3 months after hospitalization for self-poisoning. Ann Gen Psychiatry 2012;11:10. 10.1186/1744-859X-11-10 22520705 PMC3347980

